# Surgical interventions for external snapping hip syndrome

**DOI:** 10.1007/s00167-020-06305-w

**Published:** 2020-10-16

**Authors:** Filippo Randelli, Manuel Giovanni Mazzoleni, Alberto Fioruzzi, Alessio Giai Via, Vittorio Calvisi, Olufemi Rolland Ayeni

**Affiliations:** 1 Hip Department (CAD), Gaetano Pini-CTO Orthopaedic Institute, University of Milan, Milan, Italy; 2 Unit of Orthopaedics and Traumatology, Department of Life Health and Environmental Sciences, University of L’Aquila, L’Aquila, Italy; 3 Department of Orthopaedics and Traumatology, San Camillo-Forlanini Hospital, Rome, Italy; 4 Division of Orthopaedic Surgery, McMaster University, Hamilton, ON Canada

**Keywords:** External snapping hip, Coxa saltans, Greater trochanter pain syndrome, Endoscopic surgery, Hip arthroscopy

## Abstract

**Purpose:**

Snapping hip is a common clinical condition, characterized by an audible or palpable snap of the hip joint. When the snap is perceived at the lateral side of the hip, this condition is known as external snapping hip or lateral coxa saltans, which is usually asymptomatic. Snapping hip syndrome (SHS) refers to a painful snap, which is more common in athletes who require increased hip range of motion. The aim of this article is to review the most common endoscopic techniques for the treatment of ESHS, as well as their results and limitations.

**Methods:**

This is a review of the current literature of endoscopic surgical procedures and of the results of the treatment of external snapping hip syndrome.

**Results:**

The pathogenesis of SHS is mechanical. The initial treatment attempt is conservative, and usually provides good results. Patients who do not respond to conservative management are candidate for surgery. The endoscopic release of the ilio-tibial band or the endoscopic release of the femoral insertion of the gluteus maximum tendon is the most popular technique.

**Conclusion:**

Endoscopic techniques provide fewer complications compared to open surgery, a lower recurrence rate and good clinical outcomes. More comparative studies with a longer follow-up are required to adequate evaluate the full role of endoscopic techniques in periarticular hip surgery.

**Level of evidence:**

Level V.

## Introduction

Two types of snapping hip have been described, lateral (external) and medial (internal). An audible snapping hip is asymptomatic in most cases, while in a few patients, the snap may become painful, and this condition is known as snapping hip syndrome (SHS). The most common cause of a painful snapping hip is the external (external snapping hip syndrome—ESHS), which is associated with a characteristic noise, due to the flipping of the ilio-tibial band (ITB) over the greater trochanter. External snapping hip or lateral “coxa saltans” was first reported by Perrin in 1859, and then was popularized by Nunziata and Blumenfeld at the middle of the twentieth century [7]. The prevalence in general population is not well defined, as most of cases are asymptomatic, however, it has been reported to occur in up to 10% of the general population [4]. The prevalence of a painful snap is higher in individuals involved in selected activities and who require extreme hip ROM, such as ballet dancers, runners, and soccer players [11].

The aim of this article is to review the most common endoscopic techniques for the treatment of ESHS, as well as their results and limitations. Then, we reported our approach to managing this condition.

## State of the current evidence

### Pathogenesis

The ITB originates from the tensor fasciae latae anteriorly and the gluteus maximus posteriorly, it runs down on the lateral aspect of the thigh, and it inserts onto the tibial Gerdy’s tubercle [1]. It has a firm insertion to the proximal femur at the linea aspera, sharing some fibers with the gluteus maximum tendon. Also, some fibers insert onto the lateral femoral epicondyle and lateral border of the patella distally [12]. The ITB acts as a tendon of both the tensor of the fasciae latae and the gluteus maximus, stabilizing the hip during abduction and walking. The ITB is tightened by the contraction of these muscles during hip flexion, abduction, and extension. The ESH is caused by the ITB flipping back and forth across the greater trochanter due to a thickening of the posterior portion of the ITB or the anterior border of the gluteus maximus [12]. During hip flexion, the ITB and the anterior margin of the gluteus maximus glide gently anteriorly over the lateral surface of the greater trochanter. If thickened, they rub over the greater trochanter, giving rise to a snapping sound. Snapping may also occur during hip extension while the ITB and the anterior margin of the gluteus maximus muscle are moving posteriorly over the greater trochanter [12].

### Treatment of external snapping hip syndrome

The treatment of ESHS is first conservative, focusing on improving pain, flexibility and equalizing the limb length discrepancy if needed. The management includes rest, avoiding movements that provoke the snap, and reduction of the sport activities. A stretching program specific to the ITB and the iliopsoas muscles is indicated, and local corticosteroids injections and physical therapies, as laser therapy and extracorporeal shockwave therapy ESWT, can be useful to manage the pain [3]. The conservative treatment should be continued for at least 6 months [8]. Then, if the patient does not respond to a well-conduced conservative program, surgery is indicated. The goal of surgical treatment is to release the contracted ITB to resolve the snap. Many different surgical techniques have been described, both open and endoscopic, but there is still no consensus on the gold standard procedure. Current literature shows that endoscopic procedures are superior in terms of complication profile, recurrence rate, aesthetic results and patient’s satisfaction [4]. The most common endoscopic techniques for the treatment of ESHS are diamond-shaped ITB release over the greater trochanter and the release of the femoral insertion of the gluteus maximus tendon [5, 8, 9]. To provide a better comprehension of the different techniques described in literature, we divided the treatment options into two groups according to the surgical approach and strategy: inside–out or outside–in. Finally, the author’s preferred approach (Polesello Technique) is described.

### Outside–in technique

The endoscopic ITB release was first described by Ilizaliturri in [6]. This is an outside–in technique designed to access first peripherical the peritrochanteric space, above the ITB, and then to develop it by creating a diamond-shape defect on the ITB, directly above the greater trochanter. The patient can be positioned in lateral position on a standard surgical table, or in supine position on a traction table.

**Fig. 1:**
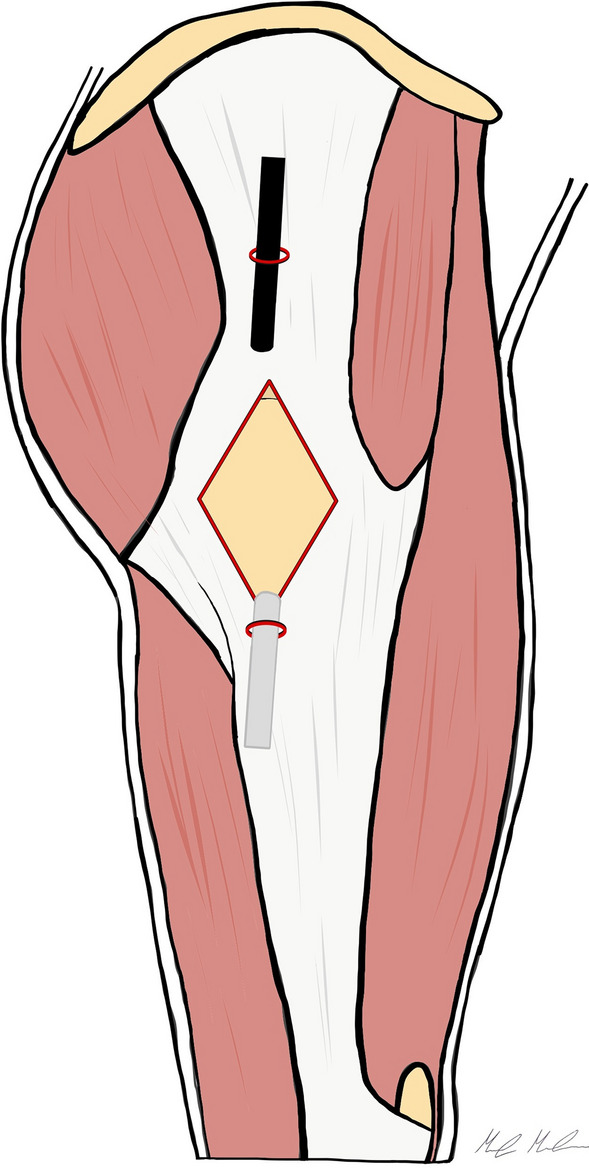
The picture shows the Ilizaliturri’s ITB release technique. A diamond-shaped defect is created over the greater trochanter to release the ilio-tibial band

The greater trochanter is outlined on the skin as the main landmark for the two endoscopic portals aligned with the axis of the femur. The inferior trochanteric portal (ITP) is located approximately 3 cm distal to the GT. The superior trochanteric portal (STP), which is the main operative portal, can be marked approximately 3 cm proximal to the tip of the GT. The snapping area should be located between the two portals. The snap is examined and confirmed under general anesthesia before the procedure. Saline solution (40–50 mL) is injected, with an 18 gauge needle, over the GT to develop the subcutaneous space. An arthroscopic 4.5 mm cannula with a blunt obturator is introduced through the ITP, under fluoroscopic guidance, to develop the space between the two portals over the ITB. Then, a 30° arthroscope is introduced with low-pressure water inflow. The STP is established under direct visualization. The subcutaneous tissue is dissected with a curved shaver blade to allow for a clear identification of the ITB. With a radiofrequency hook probe (RF), introduced from the STP, the first longitudinal 4–6 cm retrograde transecting incision of the ITB is performed, starting from the ITP in proximal direction, dividing the ITB in two components: anterior (AITB) and posterior (PITB). The greater trochanteric bursa (GTB) is visible and the water pump pressure can be increased as needed. With the RF, two 2 cm perpendicular transverse cuts are performed, starting at the middle of the longitudinal cut, with an anterior and posterior direction. The posterior transverse cut is particularly important because the snapping area is mostly located on the posterior part of the ITB, and this cut should be carried out until the snapping is resolved with while manipulating the limb. Resection with a shaver results in 4 flaps and a diamond-shape defect in the ITB (Fig. 1). Now, the hip movements are tested under direct endoscopic visualization, and the GT should move within the defect without snapping. Finally, the GTB can be easily removed through the diamond-shape defect and the abductor tendons are inspected for tears.

**Fig. 2:**
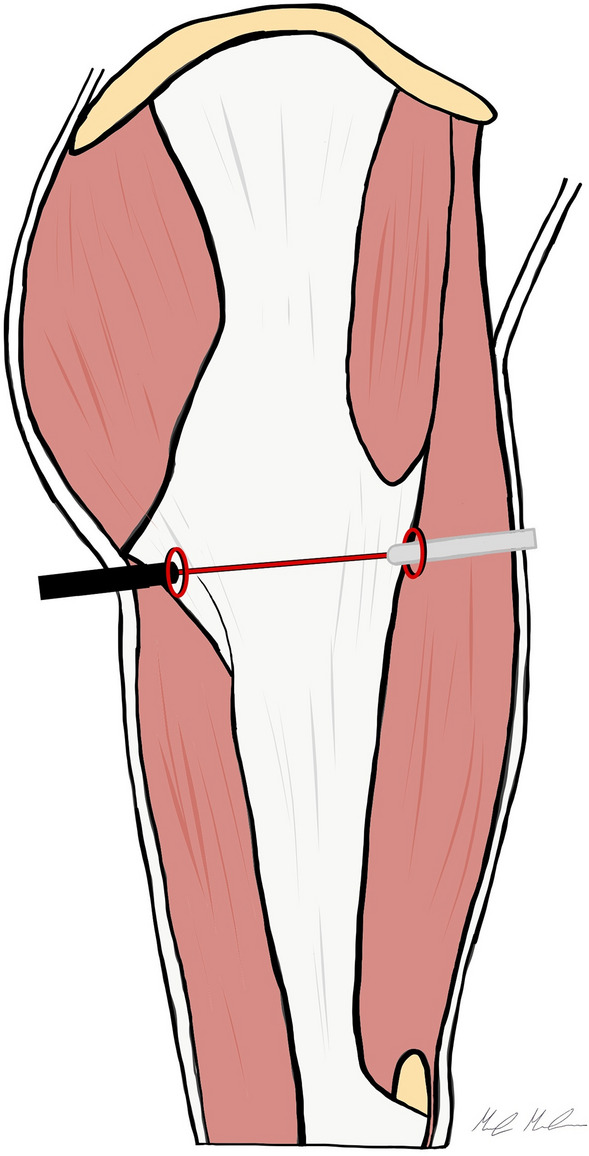
Endoscopic outside-in ITB release according Zini et al. The arthroscopic portals are created 1–2 cm anteriorly and posteriorly the femoral shaft, just below the greater trochanter, and a complete horizontal cut of the ITB is performed

A variation of this technique has been described by Zini et al. [16] with the patients placed in lateral decubitus position. Two arthroscopic portals are created perpendicular to the axis of the femur at the level of the snapping tract of the ITB, 1–2 cm anterior and posterior to the respective edges of the femoral shaft. With the 30° arthroscope through the posterior portal, and operative instruments in the anterior portal, a complete horizontal cut is performed from the anterior to the posterior edge of the ITB (Fig. 2). The authors recommended a partial release of the gluteus maximus anterior margin insertion into the ITB to minimize symptom recurrence.

### Inside–out technique

Voos et al. [14] described a different technique which allows one to perform the ITB release directly underneath it. The peritrochanteric space access is obtained, under fluoroscopic guidance, with a cannula through the anterior portal, placed 1 cm lateral to the anterior superior iliac spine with the leg in full extension and 10°–15° of internal rotation. The cannula is directed posteriorly and then slides between the ilio-tibial band and the greater trochanter to develop the operative space.

**Fig. 3:**
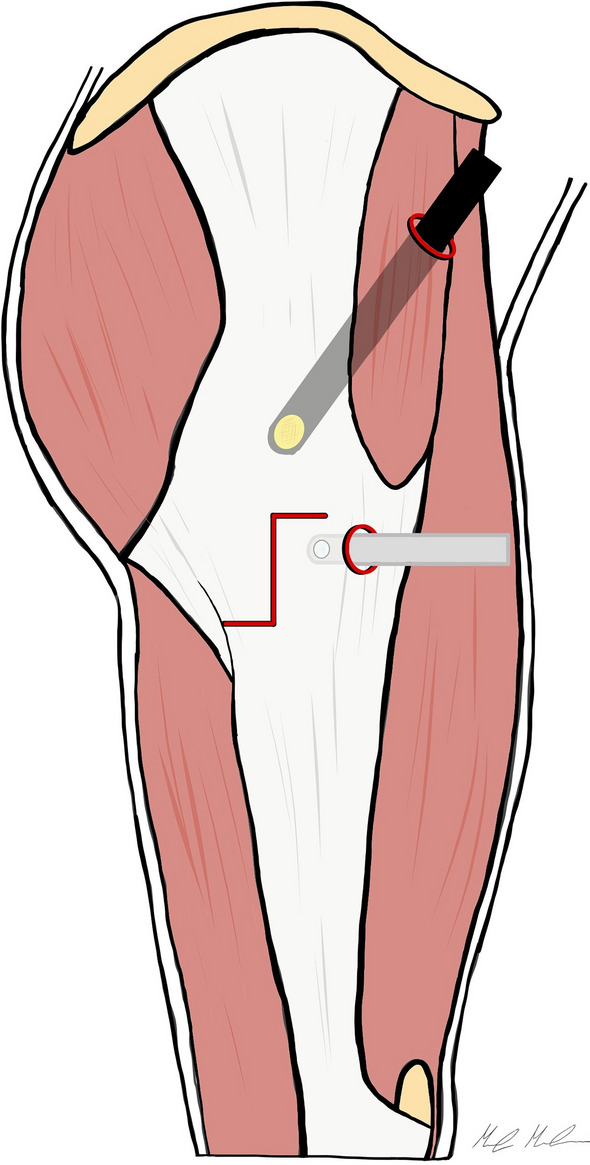
Endoscopic inside-out ITB release according Voos et al. Two arthroscopic portals are created, the anterior portal 1 cm lateral to the anterior superior iliac spine, and the operative portal, below the tip of the greater trochanter. A z-shaped incision of the ITB is performed starting beneath the fascia

The operative portal is placed midway between the tip of the greater trochanter and the vastus tubercle, approximately 4 cm distal from the GT tip, along the posterior one-third of the greater trochanteric midline. A 70° arthroscope is introduced through the anterior portal and directed distally. The peritrochanteric space is typically distended with 50–70 mmHg of pressurization which allows the visualization of the gluteus maximus insertion to both at the linea aspera and the posterior border of the ITB. The insertions can be palpated, and the bursa cleaned from this area with a motorized shaver. The ITB release is performed, with RF, along the posterolateral portion of the greater trochanter, beginning from the vastus tubercle insertion and extending to the tip of the greater trochanter in a z-shaped fashion (1 cm anterior, 3 cm distal, and 1 cm posterior) with slight variations, to include in the defect the fibers under the greatest amount of tension (Fig. 3).

**Fig. 4:**
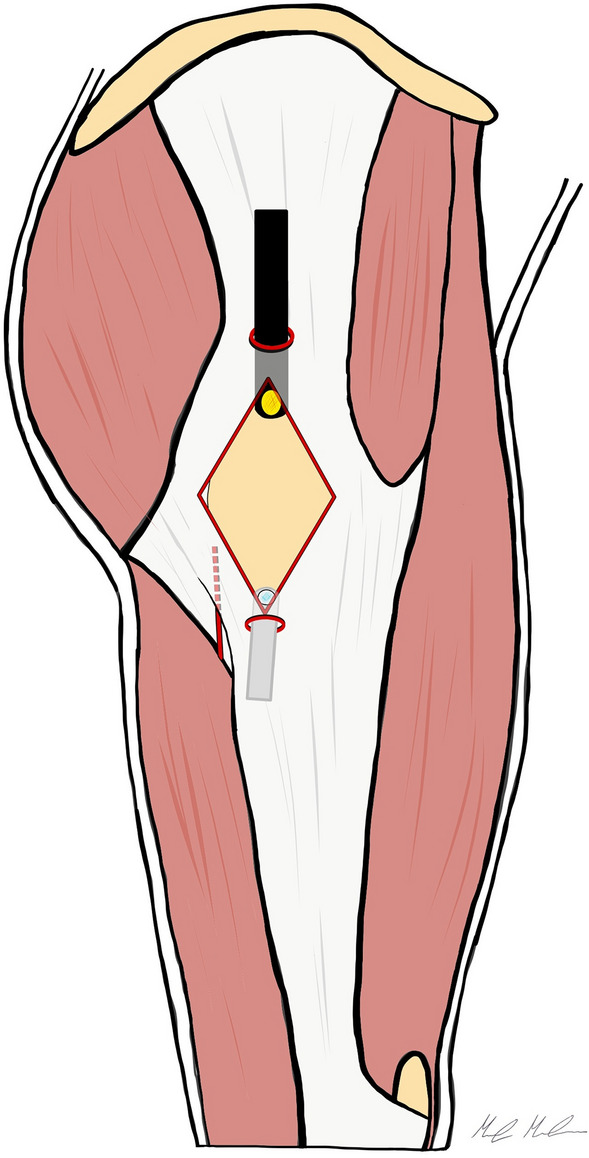
This picture shows the inside-out diamond-shaped ITB release according to Yoon et al. The two arthroscopic portals are placed anteriorly and posteriorly the border of the greater trochanter. The peritrochanteric space is developed under the ITB, and the diamond-shaped ITB release is performed

In 2014, Yoon et al. [15] reported clinical results of the endoscopic ITB and gluteal sling release. Their technique is performed with the patient positioned in lateral decubitus. The two arthroscopic portals are located on the superior and inferior extents of the greater trochanter, aligned with the femoral axis. A trocar is inserted through the inferior trochanteric portal, 1 cm below to the vastus lateralis ridge and positioned between the GT and ITB. The space between these structures is developed by gently moving the trocar for blunt dissection and maintained by injecting 40 mL of saline solution. The arthroscope is introduced through the superior trochanteric portal directly beneath the ITB allowing the evaluation of the peritrochanteric space. The ITB release is performed, in–out, with a diamond-shaped defect on the ITB and a trochanteric bursectomy is completed (Fig. 4). Finally, an additional gluteal sling release can be performed, through a transverse cut with RF, at the insertion of the gluteus maximus tendon, if needed.

#### The Author’s preferred approach (Polesello technique)

The endoscopic release of the gluteus maximum tendon insertion at the linea aspera is our preferred technique for the treatment of ESHS. It was first described by Polesello et al. in 2013 [9].

**Fig. 5:**
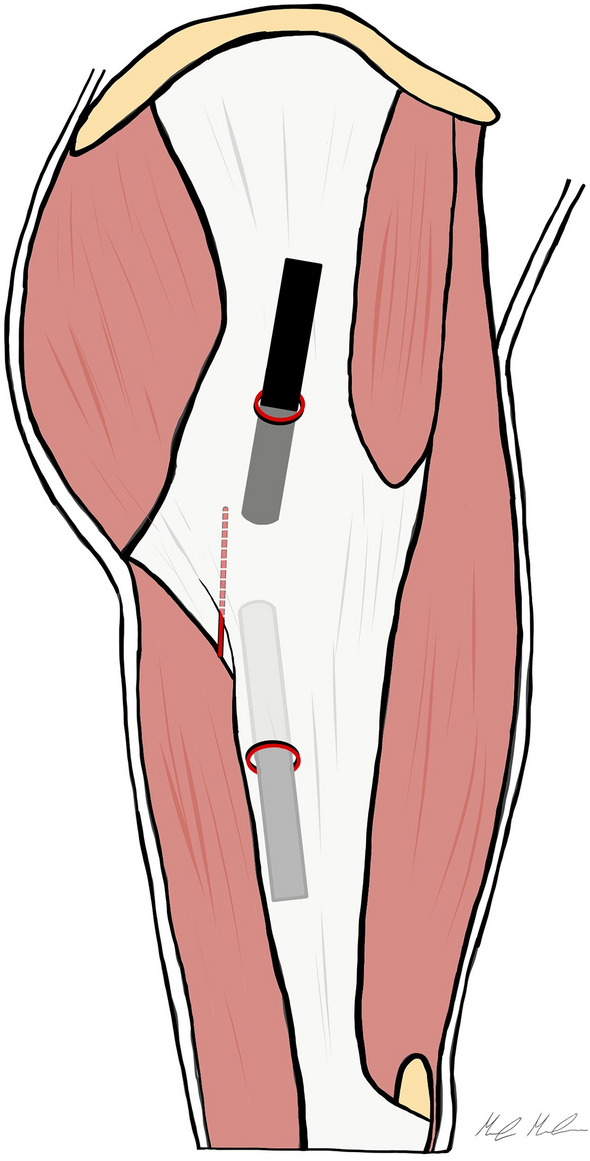
The Polesello technique. The superior trochanteric portal and the distal anterolateral accessory portal are performed. The peritrochanteric bursa is removed and the peritrochanteric space is developed until the ITB and the gluteus maximus tendon femoral insertion are clearly identified. Then, the tenotomy of the gluteus maximus tendon is performed. Dashed line: gluteus maximus tendon

**Fig. 6:**
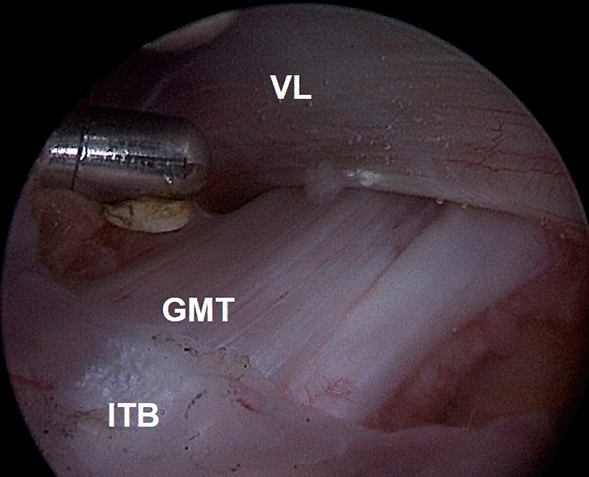
The Polesello technique. The gluteus maximus tendon is identified. It appears as a large flat tendon nearly perpendicular to the long axis of the femur. *GMT* gluteus maximus tendon, *VL* vastus lateralis, *ITB* ilio-tibial band

**Fig. 7:**
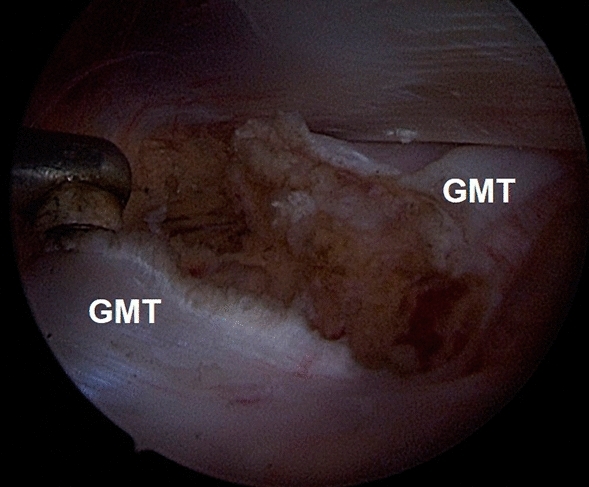
The Polesello technique. The complete resection of the gluteus maximum tendon is performed with RF probe. *GMT* gluteus maximus tendon

We perform the procedure with the patient supine on a fracture table, but without traction. Two endoscopic portals are utilized, the superior trochanteric portal (STP) and the distal anterolateral accessory portal (DALA). The STP is located 2 cm anterior and 4 cm superior to the tip of GT. A needle is introduced under fluoroscopic guidance, from the skin mark with a 45° angle in distal direction, till the greater trochanter is reached. To develop the space under the ITB, 100 ml of saline solution is injected over the prominence of the GT. Then, a standard 30° arthroscope is introduced in the peritrochanteric space, and a low-pressure water inflow maintains the space between the trochanter, the vastus lateralis and the ITB. We preferred to use a 30° arthroscope to work in the extra-articular compartment. The DALA is performed under direct visualization, 8–10 cm below the tip of GT aligned to the axis of the femur. A shaver blade is introduced through the DALA and the ITB, the trochanteric bursa is removed and the peritrochanteric space is developed from inside to outside until the ITB can be easily identified (Fig. 5). The gluteus maximus tendon (GMT) now appears posteriorly, below the vastus lateralis, as a large flat tendon with a perpendicularly oriented fibers relative to the long axis of the femur (Fig. 6). Once identified, the GMT insertion on the linea aspera, is released with RF until the tendon gap is visible (Fig. 7). Finally, the resolution of the snapping is tested dynamically under endoscopic view.

The patient is usually discharged the day after of the surgery. Full weight-bearing and full ROM of the hip are allowed as tolerated. Passive and active mobilization of the hip is started the day after the procedure, as well as isometric strengthening of quadriceps and gluteus muscles. Stationary bicycling starts twice a day for 15 min from the day after surgery. A rehabilitation period of 3 months is recommended, focusing on ROM, stretching, strengthening, core stability and proprioceptive exercises. The return to the full activities is allowed usually after 3 months, when the patients have recovered the full hip ROM strength and flexibility without pain.

## Result

There are a limited number of studies [6, 9, 13, 15, 16], which are usually retrospective, with inherent limitations in study design and sample size (Table 1). Although with several limitations, these studies showed that endoscopic techniques are safe and reproducible, and the outcomes are encouraging. Furthermore, a lower recurrence of the snap and complications rate have been reported compared to open surgery.

**Table 1:** The results of endoscopic treatments for external snapping hip syndrome have been reported

Authors and year	Patients	Technique		PROMs-preop	PROMs-postop	% Rec	Complications
		ITB release	Polesello				
Ilizaliturri, 2006	11 (12 hips)	Out–in		WOMAC 81	WOMAC 94	9%	Mild painless snapping in 1 patient
Polesello, 2013	8 (9 hips)		GMT release	HHS 61.3	HHS 77.6	22%	1 patient required a revision surgery 1 patient presented with a mild ischium snapping and pain
Zini, 2013	15	Out–in		VAS 5.5 Tegner 7.6	VAS 0.5 Tegner 7.6	0	40% of patients slight pain with strenuous exercise
Yoon, 2014	7 (10 hips)	Inside-out		VAS 6.8 mHHS 68.2	VAS 0.2 mHHS 94.8	0	Mild pain in 1 patient
Shrestha, 2017	248 (477 hips)	Out–in		–	–	0	None

*PROMs* patients reported outcome measures, *Rec* recurrence, *ITB* ilio-tibial band, *GMT* gluteus maximum tendon

Ilizaliturri et al. treated 11 patients (12 hips) with good outcomes. They reported no complication, but a patient presented with mild snapping without pain at 2 years of follow-up [6]. Zini et al. [16] evaluated a series of 15 patients who underwent an arthroscopic ITB release. After a mean 34-month follow-up (range 12–84 months), they reported no intraoperative or postoperative complications. Yoon et al. retrospectively assessed their patients who underwent a similar arthroscopic release (*n* = 10 hips) [15]. No complications were reported after a mean follow-up of 19 months (range 12–33 months). Since most of the studies were small case series with short-term follow-up, the real prevalence of complications and results needs more robust evaluation. The most common complication described after the endoscopic release of ITB is the recurrence of the snapping symptoms, which is considered a failure of the treatment. However, the recurrence rate is lower compared to open surgery [10, 13]. The sciatic nerve is in close proximity to the posterior margin of the greater trochanter, and it is at risk during peritrochanteric space endoscopy [5]. However, no complications regarding injury to the sciatic nerve have been reported up today [13]. Another concern about the ITB release is that it may cause a deformity of the shape of the lateral thigh, and an overload to the contralateral abduction mechanism, sometimes without solving the cause of the ITB compression.

**Fig. 8:**
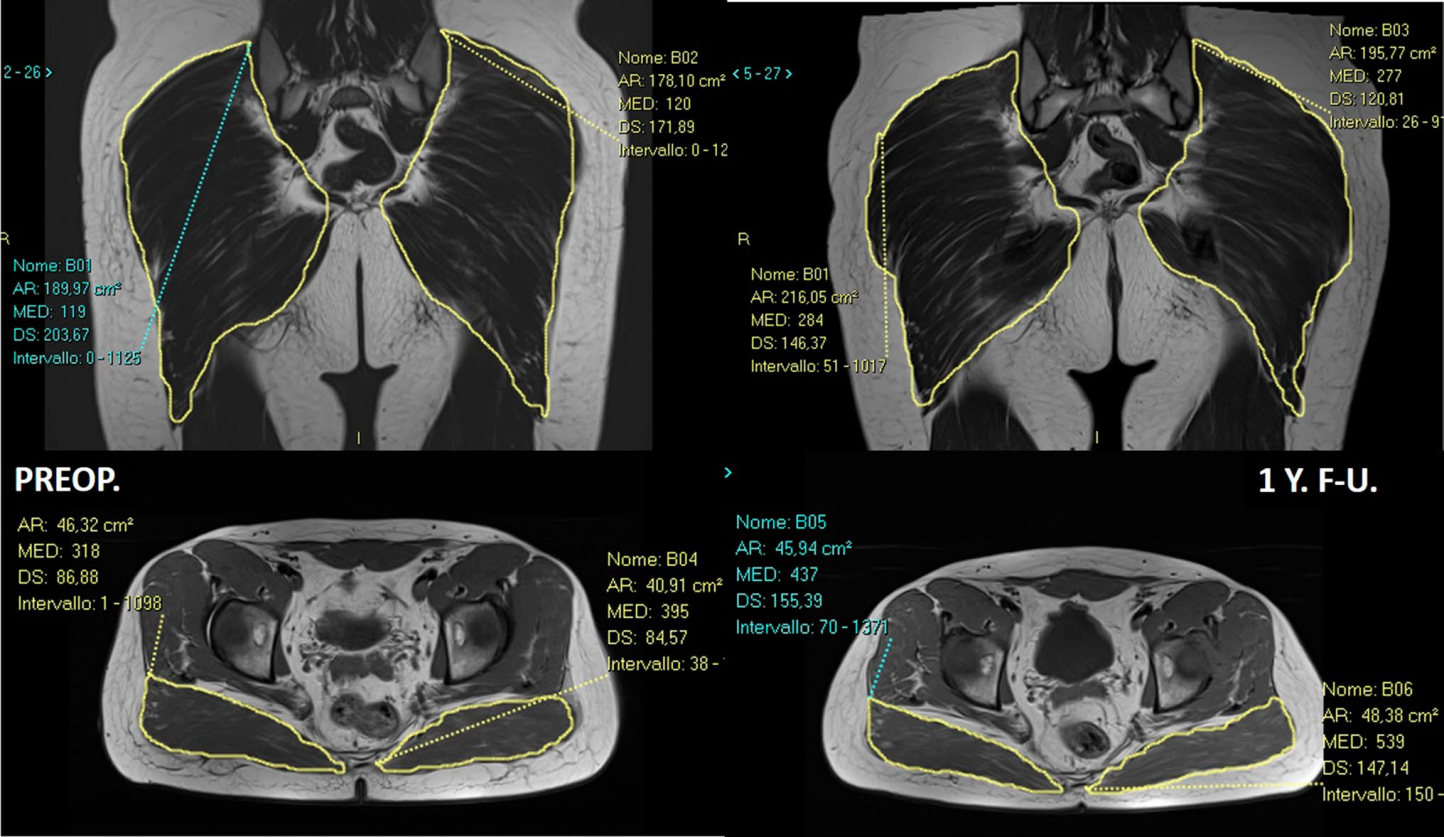
This is the MRI of a 31-year-old boy, who was operated for a femoro-acetabular impingement syndrome and external snapping hip syndrome of the right hip. Once the intra-articular procedures have been performed, the endoscopic release of the gluteus maximus tendon have been done. At 1-year follow-up, the patient was pain free, with no recurrence of the snap. The MRI shows that there are no significant differences in the cross-sectional area of the gluteus maximus muscle between the two sides, nor a significant fatty infiltration of the operated side at 1 year from surgery

The endoscopic release of the femoral insertion of the gluteus maximus tendon is based on the hypothesis that the ITB, the gluteus maximum (GM) and the tensor fascia late (TFL) muscles work as a single functional complex, as they share fibrous bundles and common insertions [2]. It was designed to reduce the pressure over the GT preserving the ITB, without compromising of the lateral complex. Polesello et al. reported on nine hips (8 patients), at a minimum of 22 months’ follow-up [9]. The snap and pain were resolved in 7 hips, while one patient required revision surgery for recurrent symptoms. At the final follow-up, all patients returned to their preoperative activity level, and no one complained weakness of the operated limb. However, few studies are reported in literature about the Polesello technique, in particular about the long-term effect of GM tendon release. The most serious concern about this technique is the residual gluteal hypotrophy and asymmetry compared to the contralateral side. From our experience, the MRI at 1-year follow-up, showed a mild hypotrophy only in few patients but minimal strength impairment of the GM muscle compared to the contralateral healthy side (Fig. 8). However, MRI studies at longer follow-up are required.

## Conclusion

Snapping hip is a common condition, but a few cases fail in conservative care and require surgery. A variety of surgical techniques have been proposed with variable success. The endoscopic techniques provided better outcomes, less complications, and better aesthetic results than open surgery. Prospective comparative studies with a longer follow-up will help refine indications for surgical approaches to ESHS.

## Abbreviations

SHS: Snapping hip syndrome
ESHS: External snapping hip syndrome
ITB: Iliotibial band
ESWT: Extracorporeal shock wave therapy
GT: Greater trochanter
ITP: Inferior trochanteric portal
STP: Iuperior trochanteric portal
RF: Radiofrequency hook probe
AITB: Anterior ilio-tibial band
PITB: Posterior ilio-tibial band
GTB: Greater trochanteric bursa
DALA: Distal anterolateral accessory portal
GTM: Gluteus maximus tendon
ROM: Range of motion
GM: Gluteus maximum
TFL: Tensor fascia latae
